# Abdominal tuberculosis presenting as a massive lower GI bleed in a cerebral palsy patient—a case report

**DOI:** 10.1093/jscr/rjaf1029

**Published:** 2026-01-07

**Authors:** Alexander M Kravets, Dzhastyn Dkhillon, Zachary Gross, Sholom-Ber Chernyak, Eugene Tarasov

**Affiliations:** Department of Surgery, Franciscan Health Olympia Fields, 20201 South Crawford Ave, Olympia Fields, IL, United States; Resurrection Medical Center, 7435 W Talcott Ave, Chicago, IL, United States; Department of Surgery, Franciscan Health Olympia Fields, 20201 South Crawford Ave, Olympia Fields, IL, United States; Resurrection Medical Center, 7435 W Talcott Ave, Chicago, IL, United States; Department of Surgery, Franciscan Health Olympia Fields, 20201 South Crawford Ave, Olympia Fields, IL, United States; Resurrection Medical Center, 7435 W Talcott Ave, Chicago, IL, United States; Department of Surgery, Franciscan Health Olympia Fields, 20201 South Crawford Ave, Olympia Fields, IL, United States; Resurrection Medical Center, 7435 W Talcott Ave, Chicago, IL, United States; Resurrection Medical Center, 7435 W Talcott Ave, Chicago, IL, United States

**Keywords:** abdominal tuberculosis, tuberculosis, lower GI bleed, cerebral palsy

## Abstract

Abdominal tuberculosis is a rare extrapulmonary manifestation of tuberculosis (TB), one that is increasingly encountered at hospitals serving a growing immigrant population. Since the COVID-19 pandemic, the incidence of TB has continually increased. The diagnostic differential often overlaps with inflammatory bowel disease, with diagnostic laparoscopy remaining the most effective modality of diagnosis. Additional diagnostic difficulties are encountered in patients unable to report symptoms. We present the case of a 52-year-old woman with cerebral palsy who presented initially with pulmonary symptoms and later developed massive lower gastrointestinal bleeding. Conservative management was attempted with angioembolization of a bleeding cecal mass on computed tomography. The patient initially improved and then decompensated after several hours. The patient then underwent emergent exploratory laparotomy for resection of a bleeding ileocecal mass which was found to be abdominal tuberculosis on pathology.

## Introduction

Public health measures and effective chemotherapy have largely improved overall outcomes in patients with tuberculosis (TB). Although the incidence of tuberculosis decreased in the United States over the course of the 20th century, a resurgence has occurred due to the advent of the human immunodeficiency virus (HIV) and an increase in immigration [1]. Moreover, there has been a spike in TB cases since the COVID-19 pandemic.

Tuberculosis remains largely manageable with antituberculosis chemotherapy; however, extrapulmonary manifestations of tuberculosis continue to contribute to significant morbidity and mortality.

Extrapulmonary manifestations of tuberculosis are often difficult to diagnose and manage. In cases of abdominal tuberculosis, patients present with nonspecific complaints, such as abdominal pain, systemic symptoms, and—occasionally—gastrointestinal (GI)-related complications. Abdominal tuberculosis is often diagnosed intraoperatively and patients are effectively managed with anti-TB chemotherapy [1–3].

## Case presentation

A 52-year-old woman was brought by her family from home to the emergency room for fever, shortness of breath, and congestion over the last 2–3 days. Her sister normally provides care and denied any medical history or prior hospitalization in the past 25 years. The family was noted to have immigrated from the Indian subcontinent. The patient was noted to be cachectic, weighing only 67 pounds. Her sister claimed the patient was normally ambulatory and feeding herself with assistance. The patient was nonverbal at baseline.

The vitals included a maximum temperature of 101.8°C, tachycardia which responded to fluid boluses, hypotension, intermittent desaturation to the low 80s, coarse lung sounds, and benign abdominal exam. Labs included elevated sodium at 155, potassium at 2.8, bicarb at 20, albumin < 1.5 g/dl, lactate at 3.3, a WBC count of 9.1 cells/mm^3^, and hemoglobin at 10.9 g/dl. Chest X-ray demonstrated coarse interstitial markings. The patient was then started on ceftriaxone and azithromycin for possible secondary pneumonia and admitted to the ICU.

**Figure 1:**
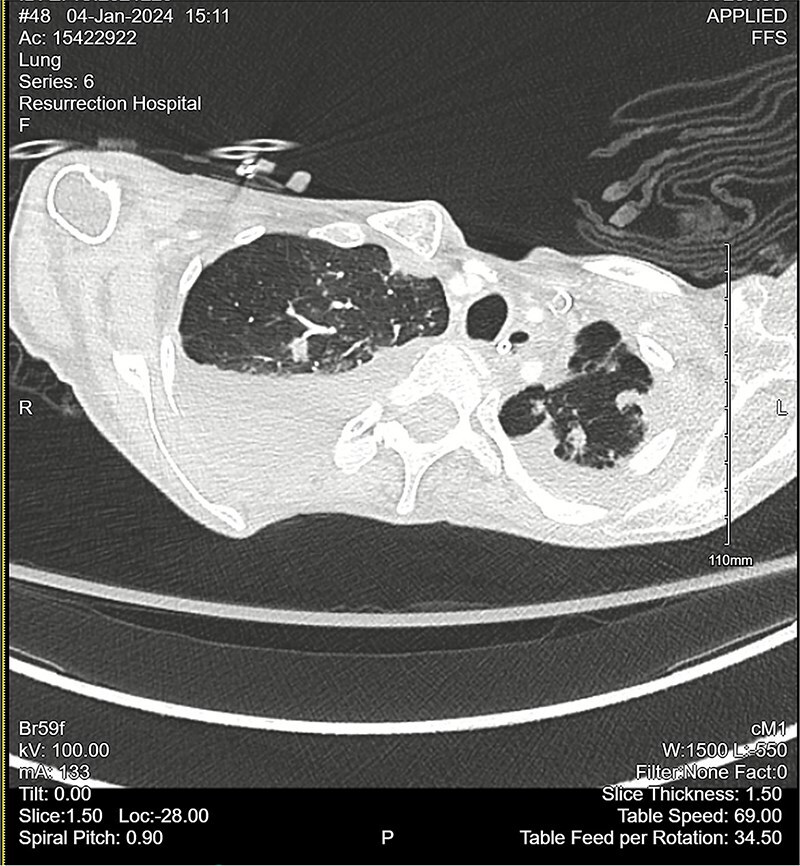
CT chest angiogram with axial cross-section demonstrating bilateral pulmonary nodules and scattered ground-glass lesions in the upper lobes. No signs of embolism in the pulmonary arterial segments. Reduced lung volumes secondary to bilateral pleural effusions are observed.

**Figure 2:**
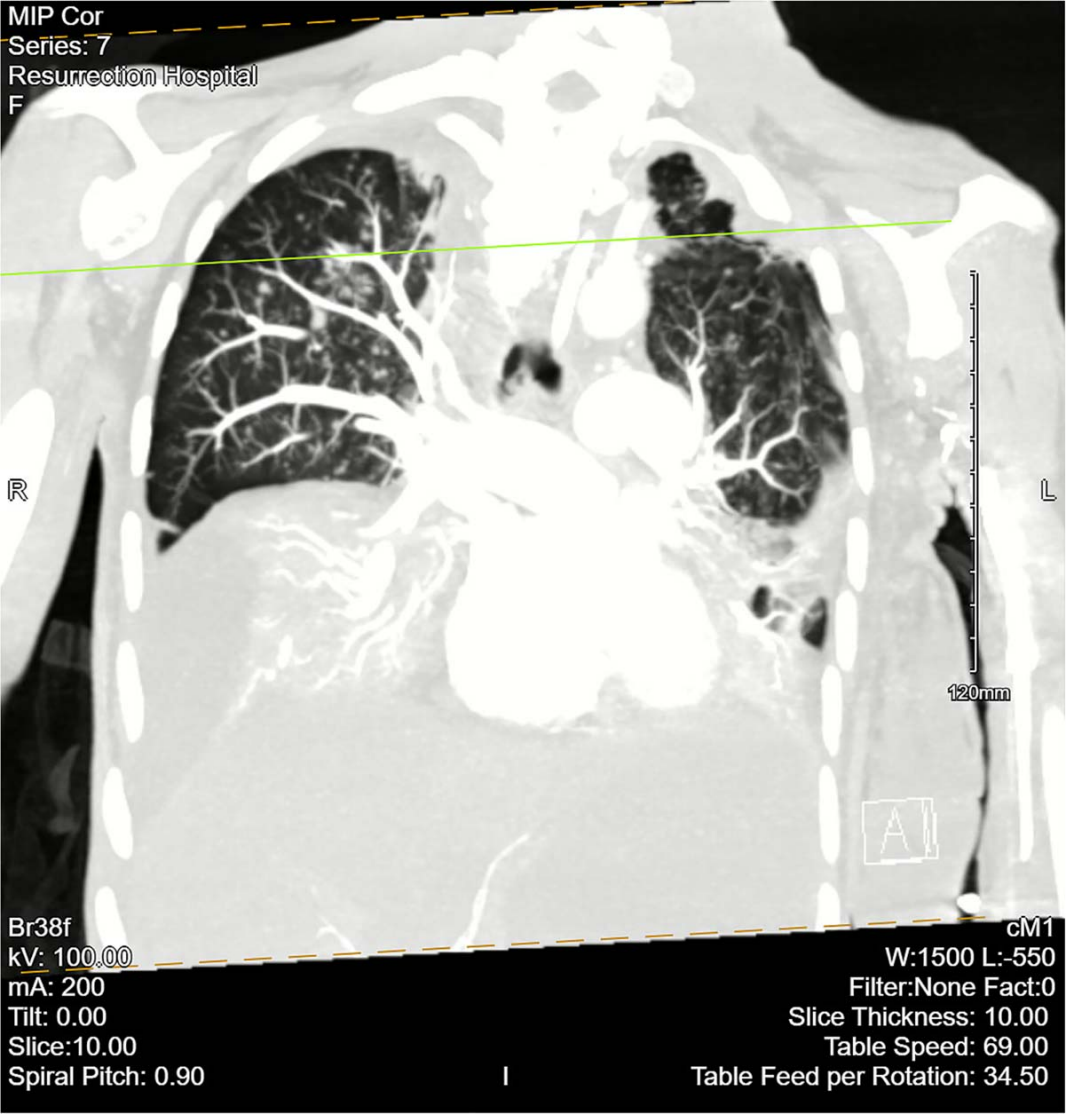
CT chest angiogram with a coronal cross section. Bilateral pleural effusions with atelectasis are observed. Ground-glass opacities with scattered nodules are present in the upper lobes. Lung volumes are reduced. There are no signs of embolism in pulmonary arterial segments.

Computed tomography (CT) angiogram of the chest demonstrated numerous small nodular and patchy opacities throughout the upper lobes ranging from punctate to 1.2 cm in diameter (Figs 1 and 2). Additionally, bilateral pleural effusions with extensive atelectasis were also observed.

The patient required intubation and vasopressor support. Later, the patient developed profuse nonbloody diarrhea with increasing abdominal distention. The GI team was consulted and recommended an upper gastrointestinal contrast study for evaluation of gastric outlet obstruction, which was negative. The patient additionally underwent an infectious workup, with a *Clostridoides difficile*, GI pathogen, and ova parasite panel, which were also negative.

**Figure 3:**
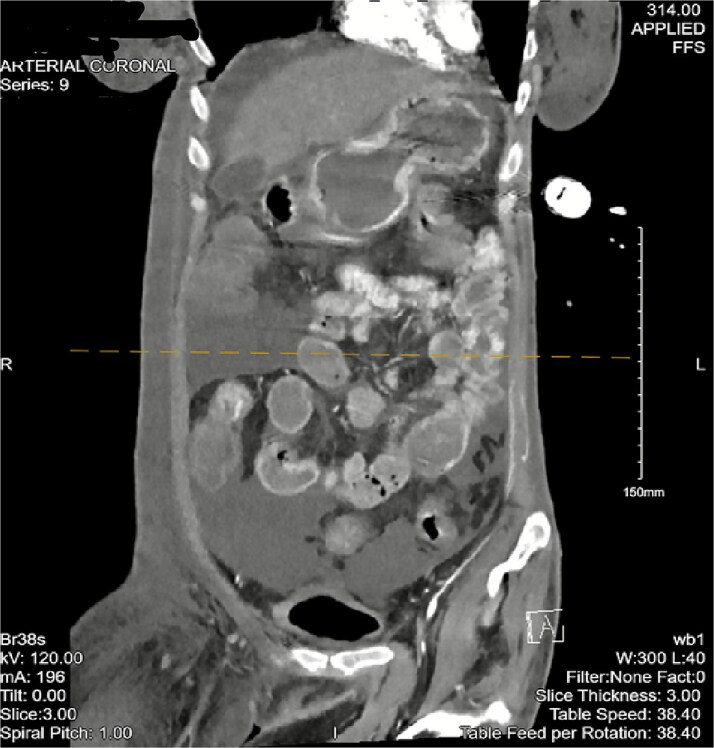
CT angiogram of the abdomen and pelvis—arterial phase with a coronal cross section. Patchy contrast extravasation is observed in the ileocecal region with adjacent distal wall-thickening. Distended loops of small bowel with mesenteric fat stranding are observed. Significant abdominopelvic free fluid is seen, correlating with ascites. Enlarged mesenteric lymph nodes are observed.

**Figure 4:**
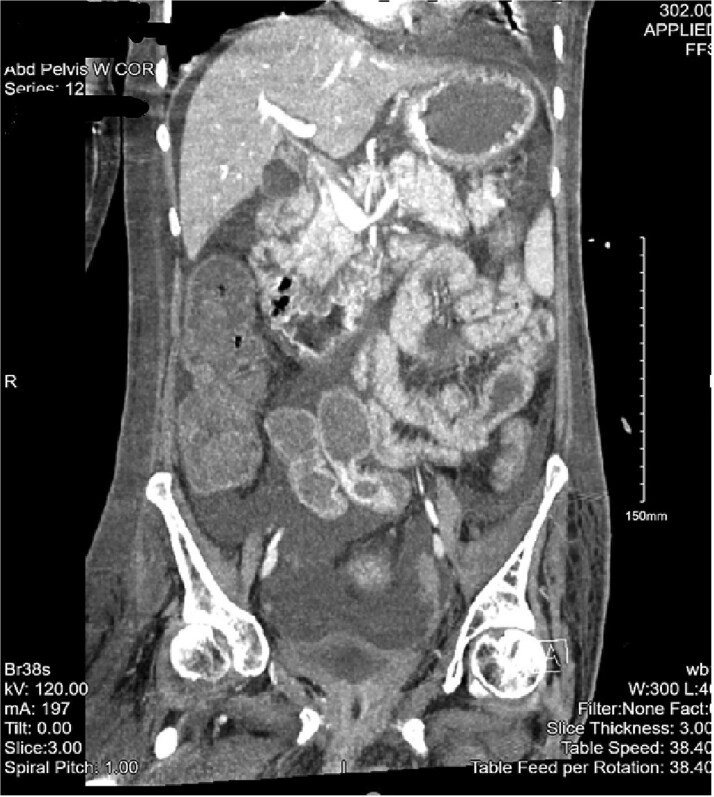
CT of the abdomen and pelvis with a coronal cross-section. Asymmetric wall thickening is observed in the cecum and ascending colon with adjacent fat stranding. Proximal dilation of small bowel loops with mesenteric stranding is also seen. Significant abdominopelvic free fluid is demonstrated. The CT findings correlate with mass versus inflammatory colitis. The read was addended to reflect concerns for cecal mass.

The patient then began having continuous bloody bowel movements with requirements for multiple blood transfusions. CT angiogram of the abdomen and pelvis demonstrated wall thickening of the cecum/ascending colon and distal active extravasation of contrast (Figs 3 and 4). These findings were consistent with a cecal mass. A massive transfusion protocol was initiated and interventional radiology was emergently consulted for angioembolization.

A superior mesenteric artery angiogram demonstrated bleeding from the ileocolic artery. Angioembolization of the branches was performed with coiling, with postembolization imaging demonstrating no signs of persistent contrast extravasation. Overnight, the patient initially improved but suddenly destabilized after having two large melenic bowel movements with increased abdominal distention and reinitiation of pressor support. The decision was then made to take the patient for exploratory laparotomy to resect the bleeding cecal mass.

In the operating room, a midline incision was made and deepened to the fascial layer. Significant edema with leakage of serous fluid from the tissue was encountered. After opening the abdominal wall fascia, approximately 0.5 L of ascitic fluid was aspirated. A cecal mass was visualized with an area of adhesion to the small bowel. Evaluation showed necrotic features of the bowel wall with inflammatory changes. The remainder of the colon appeared without any gross lesions or masses. A decision was made to resect what was found to be a necrotic ileocecal mass. Right hemicolectomy was performed with primary side-to-side anastomosis. The resected mass was sent for pathology.

Pathological examination of the resected ileocecal mass demonstrated caseating granulomas positive for acid-fast bacillus (AFB) staining and 12 lymph nodes with caseating granulomas also positive for AFB staining. The pathological findings were consistent with mycobacterial infection.

The patient was then suspected to have disseminated TB and underwent a subsequent workup with sputum cultures and initiation of anti-TB chemotherapy.

## Discussion

The number of reported TB cases and incidence rate in the United States increased in 2023 for the third year since 2020, surpassing pre-COVID-19 pandemic levels [4]. This represents an increase in the clinical burden of TB, predominantly affecting vulnerable patient populations. Several studies have shown a relatively high incidence of extrapulmonary disease among HIV-infected individuals and immigrant populations from endemic regions [1].

The pathogenesis of abdominal TB derives from gastrointestinal invasion, triggering necrotizing granulomatous inflammation. Such inflammation can lead to ulceration, bleeding, and perforation. Areas containing dense areas of proliferative lymphoid tissue and M-cells, such as the terminal ileum, are particularly susceptible to invasion [2]. Although TB may manifest in any location throughout the luminal gastrointestinal tract, the ileocecal region is most often involved [2].

The differential diagnosis includes inflammatory bowel disease, neoplasm, and infectious etiology. Characteristic CT imaging findings pointing to abdominal TB may include ascites, scattered nodular lesions, and mesenteric lymphadenopathy with hypodense centers. Paracentesis may serve as a useful adjunct diagnostic modality. On diagnosis, initiation of an anti-TB regimen is considered the mainstay of treatment. Clinical improvement can be expected as soon as 2 weeks. Endoscopic improvement can be seen after 3 months [5]. Adjunctive endoscopic, radiologic, and surgical interventions may be employed to manage complications when necessary [2].
